# Spontaneous Multiple Cervical Artery Dissections Detected with High-Resolution MRI: A Prospective, Case-Series Study

**DOI:** 10.3390/jcm14186635

**Published:** 2025-09-20

**Authors:** Aikaterini Foska, Aikaterini Theodorou, Maria Chondrogianni, Georgios Velonakis, Stefanos Lachanis, Eleni Bakola, Georgia Papagiannopoulou, Alexandra Akrivaki, Stella Fanouraki, Christos Moschovos, Panagiota-Eleni Tsalouchidou, Ermioni Papageorgiou, Athina Andrikopoulou, Klearchos Psychogios, Odysseas Kargiotis, Apostolοs Safouris, Effrosyni Koutsouraki, Georgios Magoufis, Dimos-Dimitrios Mitsikostas, Sotirios Giannopoulos, Lina Palaiodimou, Georgios Tsivgoulis

**Affiliations:** 1 Second Department of Neurology, National and Kapodistrian University of Athens, School of Medicine, “Attikon” University Hospital, 12462 Athens, Greece; dkfoska@gmail.com (A.F.); katetheo24@gmail.com (A.T.); mariachondrogianni@hotmail.gr (M.C.); elbakola@yahoo.gr (E.B.); georgiapap22@hotmail.com (G.P.); alexandra.akrivaki@gmail.com (A.A.); stelfanou@gmail.com (S.F.); moship@windowslive.com (C.M.); pania.tsalouchidou@gmail.com (P.-E.T.); safouris@yahoo.com (A.S.); sgiannop@uoi.gr (S.G.); lina_palaiodimou@yahoo.gr (L.P.); 2 Second Department of Radiology, National and Kapodistrian University of Athens, School of Medicine, “Attikon” University Hospital, 12462 Athens, Greece; giorvelonakis@gmail.com; 3 Iatropolis Magnetic Resonance Diagnostic Centre, 15231 Athens, Greece; steflach61@gmail.com; 4 Epilepsy Center Hessen, Department of Neurology, Philipps University Marburg, 35043 Marburg, Germany; 5 Stroke Unit, Metropolitan Hospital, 18547 Piraeus, Greece; erminapapageorgiou@yahoo.gr (E.P.); andriathi@yahoo.gr (A.A.); apsychoyio@yahoo.gr (K.P.); kargiody@gmail.com (O.K.); 6 First Department of Neurology, School of Medicine, Aristotle University of Thessaloniki, AHEPA University Hospital, 54636, Thessaloniki, Greece; efrosink@gmail.com; 7 Interventional Radiology Unit, Second Department of Radiology, National and Kapodistrian University of Athens, School of Medicine, “Attikon” University Hospital, 12462 Athens, Greece; magoufisgeorge1@me.com; 8 Interventional Neuroradiology Unit, Metropolitan Hospital, 18547 Piraeus, Greece; 9 First Department of Neurology, National and Kapodistrian University of Athens, School of Medicine, “Eginition” Hospital, 11528 Athens, Greece; dimosmitsikostas@me.com

**Keywords:** cervical artery dissection, ischemic stroke, high-resolution vessel wall imaging, MR-angiography

## Abstract

**Background**: Cervical artery dissection (CAD) is a leading cause of acute ischemic stroke among young and middle-aged patients. Currently, the growing availability of high-resolution magnetic resonance imaging (MRI), particularly fat-saturated T1-weighted black-blood SPACE sequences, allows the non-invasive, rapid, and reliable diagnosis of multiple arterial dissections. **Methods**: We reported our experience from two tertiary stroke centers of patients diagnosed with spontaneous multiple cervical artery dissections, detected with high-resolution MRI, during a three-year period (2022–2025). **Results**: Among 95 consecutive patients with CAD, 11 patients (mean age: 48 ± 9 years, 6 (55%) females) were diagnosed with multiple symptomatic or asymptomatic CADs, whereas in 84 patients (mean age: 49 ± 11 years, 32 (38%) females) a single CAD was detected. In all patients, high-resolution MRI and MR-angiography were performed, whereas digital subtraction angiography (DSA) with simultaneous evaluation of renal arteries was conducted in nine patients. A history of trauma or chiropractic manipulations, intense physical exercise prior to symptom onset, recent influenza-like illness, and recent childbirth in a young female patient were reported as predisposing risk factors. Cervicocranial pain, cerebral infarctions leading to focal neurological signs, and Horner’s syndrome were among the most commonly documented symptoms. Characteristic findings in the high-resolution 3D T1 SPACE sequence were detected in all patients. Fibromuscular dysplasia and Eagle syndrome were detected in four patients and one patient, respectively. Eight patients were treated with antiplatelets, whereas three patients received anticoagulation with low-molecular-weight heparin. There was only one case of stroke recurrence during a mean follow-up period of 9 ± 4 months. **Conclusions:** This case series highlights the utility of specific high-resolution MRI sequences as a very promising method for detecting multiple CADs in young patients. The systematic use of these sequences could enhance the sensitivity of detecting multiple cervical CADs, affecting also the thorough investigation for underlying connective tissue vasculopathies, stratifying the risk for first-ever or recurrent ischemic stroke, and influencing acute reperfusion and secondary prevention therapeutic strategies.

## 1. Introduction

Cervical artery dissection (CAD) is the second leading cause of acute ischemic stroke among young and middle-aged patients [1,2]. However, simultaneous multiple artery dissections involving vessels of anterior and posterior circulation have been rarely described [3]. In approximately 75–80% of patients with CAD, a single dissection is found at initial presentation, whereas in 15–20% of patients, multiple dissections (double or even triple, or even quadruple) can be detected [4].

Simultaneous multiple cervical dissections have been associated with predisposing risk factors, including neck or brain trauma, chiropractic manipulations, intense physical exercise, recent childbirth or flu-like illness, and underlying connective tissue vasculopathies (e.g., higher prevalence of fibromuscular dysplasia) [3,5,6]. Moreover, genetic predispositions (e.g., Ehlers–Danlos syndrome) and neuroimaging findings (e.g., vessel tortuosity and findings indicative of Eagle syndrome) have been implicated in the manifestation of multiple CADs [7,8].

Clinical manifestations vary and may include focal neurological signs attributed to cerebral ischemia, either isolated or combined with neck pain and headache, and local signs such as Horner’s syndrome and isolated or multiple cranial nerve palsy, pulsatile tinnitus, and cervical radiculopathy. Pain location varies according to the artery involved [9,10,11].

Digital subtraction angiography (DSA) represents the previous “gold-standard” method for the diagnosis of cervical artery dissections [12]. Nevertheless, it has been currently replaced by the non-invasive and widely available high-resolution magnetic resonance imaging (MRI) vessel wall imaging [13]. Fat-saturated T1-weighted black-blood SPACE (sampling perfection with application-optimized contrast using different flip-angle evolutions) sequences can easily diagnose different characteristics of a cervical dissection, such as mural hematoma, luminal flap, false lumen, long tapered stenosis, and dissecting aneurysm, providing valuable, clinically relevant information [14,15,16].

Multiple dissections have been associated with an increased risk of subsequent ischemic stroke in recent studies [17]. Nevertheless, evidence regarding the safety and efficacy of anticoagulation versus antiplatelet therapy for stroke prevention in these patients remains inconclusive [18,19,20,21].

In view of the former considerations, we sought to determine the prevalence of baseline characteristics, clinical manifestations, neuroimaging findings, and potential underlying etiologies in patients with multiple CADs from two tertiary care stroke centers during a three-year period. We aimed to underscore the value of high-resolution MRI vessel wall imaging in the comprehensive evaluation of extracranial and intracranial vasculature, as well as in identifying symptomatic and asymptomatic dissections.

## 2. Materials and Methods

The data that support the findings of the present study are available from the corresponding author upon reasonable request.

### 2.1. Participants

Patients with detected multiple (≥2) CADs were identified in a consecutive registry of 95 patients with CAD and were recruited prospectively between June 2022 and June 2025 at the Second Department of Neurology of National & Kapodistrian University of Athens at “ATTIKON” University Hospital in Athens and the Stroke Unit of Metropolitan Hospital in Piraeus. The patients presented either at our stroke units or at our outpatient stroke clinics. Detailed medical and patient history intake and thorough clinical examination were performed on all patients of this registry to identify CAD-associated clinical signs. Patients without available brain MRI or with missing sequences, including fat-saturated T1-weighted black-blood SPACE sequences, were excluded from this study. Moreover, patients who did not provide consent to participate in the current study were also excluded.

CADs were categorized as spontaneous when occurring spontaneously or associated with an effort or minor trauma [22]. Vascular or other potential risk factors and precipitating events for CAD were assessed and reported as previously described in detail [23,24].

We primarily abstracted data on demographic variables, vascular risk factors, medical history, and potential precipitating events. Moreover, clinical characteristics and neuroimaging findings, as well as treatments and outcomes of all included patients, were documented and further evaluated.

### 2.2. Neuroimaging Studies

For all the patients, 3 Tesla MRI scans, which included T2/Fluid Attenuated Inversion Recovery (FLAIR), T1, Diffusion-Weighted Imaging (DWI), Susceptibility-Weighted Imaging (SWI), and T1-post-gadolinium sequences, were available for the diagnosis. MR-angiography (MRA) of cervical and brain arteries, including the 3D fat-saturated black-blood 3 Tesla T1 weighted vessel wall imaging (SPACE), was also performed in all patients. Moreover, DSA was conducted by an independent interventional neuroradiologist (G.M.), evaluating the renal arteries as well. Two independent neuroradiologists (G.V. and S.L.) reviewed all MRI and MRA scans. The following markers were assessed: mural hematoma, luminal flap, false lumen, long tapered stenosis, and dissecting aneurysm.

### 2.3. Ethical Approval and Patient Consent

This study was conducted in accordance with the Declaration of Helsinki and approved by the Ethics Committee of “ATTIKON” University Hospital (Decision Numbers: ΕΒΔ 648/20 November 2020 and Decision Number: EBΔ 354/15 May 2024). All patients provided written informed consent for the publication of this study in accordance with the Declaration of Helsinki in its currently applicable form.

### 2.4. Statistical Analysis

We assessed the pooled prevalence of baseline characteristics of all patients included in the present case series. Continuous variables were presented as mean with standard deviation (SD) in the case of the normal distribution that was assessed using the Kolmogorov–Smirnov test. Continuous variables with skewed distribution were presented as median with interquartile ranges (IQR). Categorical variables were presented as the number of patients with the corresponding percentages. All statistical analyses were conducted using the R software version 2025.05.0+496 [25].

## 3. Results

Among 95 patients with carotid and/or vertebral artery dissections, we identified 11 patients (mean age: 48 ± 9 years, 6 (55%) females) with multiple CADs, whereas in 84 patients (mean age: 49 ± 11 years, 32 (38%) females) a single CAD was detected. A total of ten patients had double CAD, while in one patient three cervical vessels were simultaneously affected with CAD. Among the 23 cervical arteries affected, CAD was located in the internal carotid artery (*n* = 19) and the vertebral artery (*n* = 4). Demographic characteristics, vascular risk factors, medical history, triggering events, and outcomes are summarized in Table 1. Recent cervical trauma was documented in one patient; another patient reported symptom onset following intense physical exercise, while a female patient with multiple dissections had a history of recent childbirth via cesarean section.

Clinical manifestations are also summarized in Table 1. Focal neurological symptoms, attributed to acute or chronic ischemic lesions, were detected in eight patients, whereas three patients presented with cervicocranial or maxillofacial pain, frequently associated with ipsilateral Horner syndrome. Notably, one patient reported recurrent episodes of left-sided cervicocranial pain resembling cluster headache.

**Figure 1:**
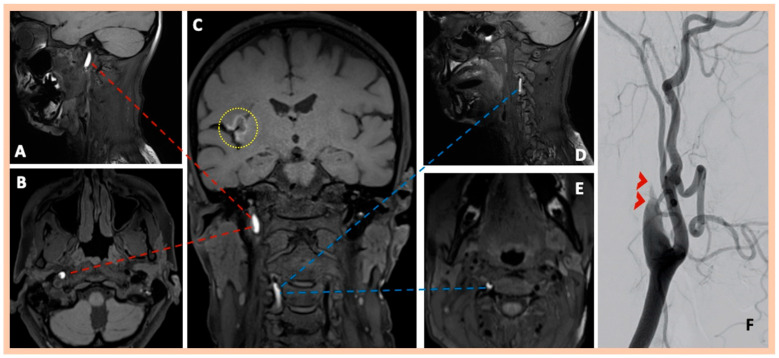
Neuroimaging findings—Patient #1. Three-dimensional fat-saturated black-blood T1-weighted high-resolution (3 Tesla) vessel wall imaging with multiplanar reconstruction, showing a characteristic (crescent-shaped), hyperintense lesion in the vessel wall of the right internal carotid artery, indicating intramural hematoma (Panels (**A**): Sagittal—(**B**): Axial—(**C**): Coronal; red dotted lines). Moreover, an asymptomatic spontaneous dissection with intramural hematoma along the distal intraforaminal (V2) segment of the right vertebral artery was detected using the same MR sequence (Panels (**C**): Coronal—(**D**): Sagittal—(**E**): Axial; blue dotted lines). The subacute right middle cerebral artery infarction is also depicted in the T1-weighted SPACE images. (Panel (**C**)—yellow circle with dotted lines). Digital subtraction angiography confirmed the right ICA dissection, revealing a “flame-shaped” occlusion (Panel (**F**)).

**Figure 2:**
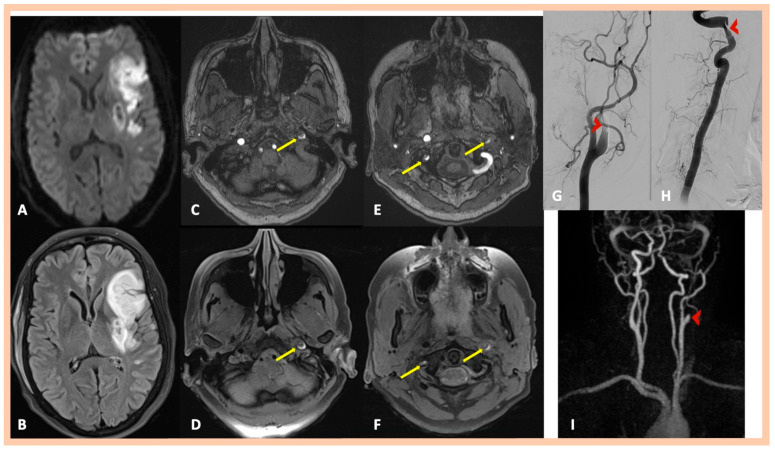
Neuroimaging findings—Patient #2. High-resolution 3 Tesla brain MRI showing a left middle cerebral artery infarction (Panel (**A**): diffusion restriction in axial DWI sequence; Panel (**B**): hyperintense signal in axial FLAIR imaging). Double lumen and intraluminal hematoma along the left ICA are depicted in Time-of-Flight (TOF) MRA (Panel (**C**)) and 3D fat-saturated black-blood T1-weighted high-resolution (3 Tesla) vessel wall imaging (Panel (**D**)), respectively. An asymptomatic spontaneous dissection with double lumen and intramural hematoma along the V3 segment of the right vertebral artery was also detected using the same MR sequences (Panel (**E**): TOF MRA—Panel (**F**): 3D fat-saturated black-blood T1 SPACE). Digital subtraction angiography confirmed the left ICA dissection, revealing a “flame-shaped” occlusion (Panel (**G**)) and also revealed focal stenosis (approximately 80%) along the V3 segment of the right vertebral artery, indicative of intramural hematoma (Panel (**H**)). MRA showing the left ICA occlusion as well (Panel (**I**)).

**Figure 3:**
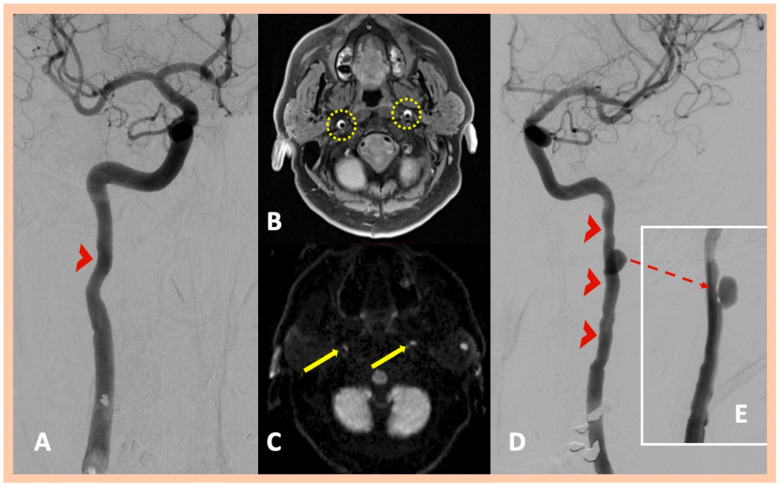
Neuroimaging findings—Patient #3. Three-dimensional fat-saturated black-blood T1-weighted high-resolution (3 Tesla) vessel wall imaging, showing crescent-shaped, hyperintense lesions in the vessel walls of the right and left ICA (Panel (**A**)), indicating intramural hematoma. Characteristic crescent- or ring-shaped lesions with DWI restriction are detected along the affected segments of the ICA bilaterally (Panel (**B**)). Digital subtraction angiography showing focal stenosis along the extracranial segment of the right ICA, indicative of intramural hematoma (Panel (**C**)), and long tapered stenosis along the extracranial left ICA (Panel (**D**)), with the presence of a dissecting aneurysm (Panel (**E**)).

**Figure 4:**
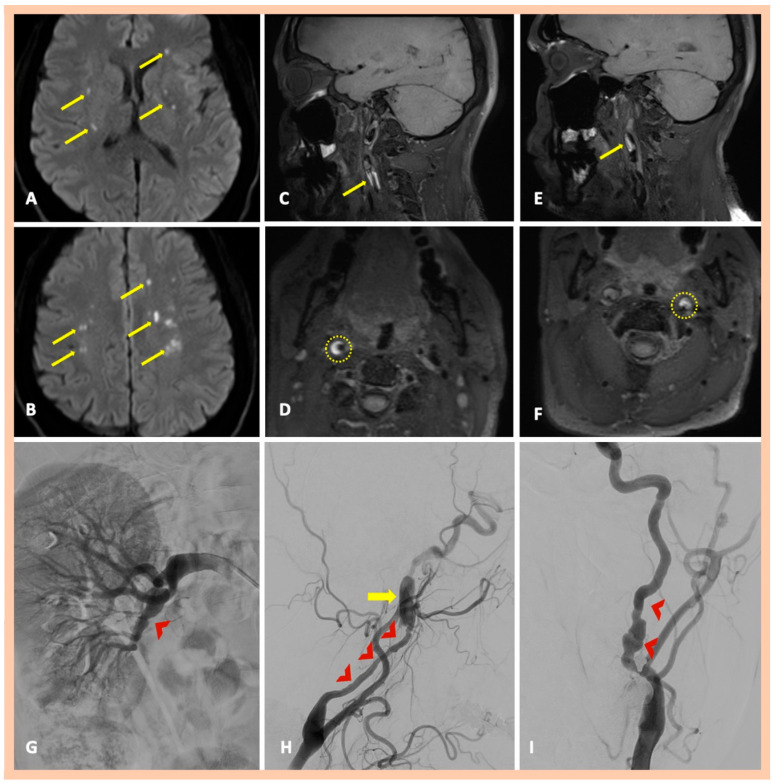
Neuroimaging findings—Patient #7. DWI sequence revealing multiple acute ischemic lesions with diffusion restriction in multiple arterial territories bilaterally (Panels (**A**,**B**)). Three-dimensional fat-saturated black-blood T1-weighted high-resolution (3 Tesla) vessel wall imaging with multiplanar reconstruction, showing crescent-shaped, hyperintense lesions in the vessel wall of the right ICA (Panels (**C**): Sagittal—(**D**): Axial) and left ICA as well (Panels (**E**): Sagittal—(**F**): Axial), indicating intramural hematoma along both arteries. DSA revealing the characteristic focal stenosis of the right renal artery (Panel (**G**)) and the characteristic “string-of-beads” appearance along the extracranial segments of the right (Panel (**H**)) and left ICA (Panel (**I**)) with the presence of a dissecting aneurysm along the distal cervical and proximal petrous artery segments of the right ICA (Panel (**H**); yellow arrow).

Brain MRI demonstrated acute or subacute ischemic infarctions within single or multiple arterial territories in six and two patients, respectively. Symptomatic CADs were revealed in MRA, whereas 3D fat-saturated black-blood T1-weighted high-resolution (3 Tesla) vessel wall imaging with multiplanar reconstruction showed asymptomatic, simultaneous CADs that had not been detected in the MRA in 10 patients (Figure 1 and Figure 2). A characteristic crescent- or ring-shaped DWI hypersignal in the affected artery was detected in three patients (Figure 3B), whereas intramural hematoma with associated long tapered stenosis was the most frequently detected abnormality in 3D fat-saturated black-blood T1-weighted imaging (Figure 4). Interestingly, no intracranial dissection was detected in our cohort.

**Figure 5:**
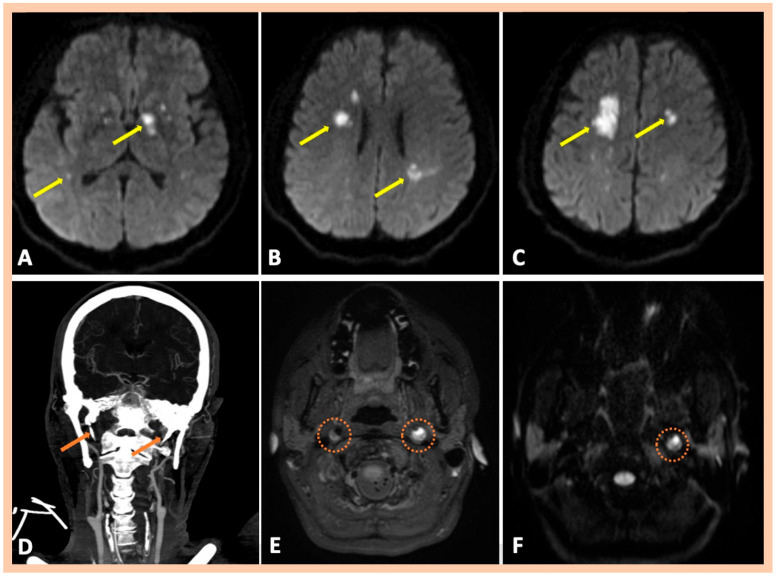
Neuroimaging findings—Patient #10. DWI sequence revealing multiple acute ischemic lesions with diffusion restriction in bilateral anterior circulation territories (Panels (**A**–**C**)). Computed tomography of the neck and brain revealed elongated styloid processes of the stylohyoid ligaments bilaterally (Panel (**D**)). Three-dimensional fat-saturated black-blood T1-weighted high-resolution (3 Tesla) vessel wall imaging showing crescent-shaped, hyperintense lesions in the vessel wall of the right ICA in the subacute phase (Panel (**E**)) and of the left ICA in the acute phase (Panel (**E**)). A characteristic crescent- or ring-shaped lesion with DWI restriction is detected along the affected segment of the left ICA (Panel (**F**)).

DSA was performed in nine patients; in two additional cases, it was recommended but not performed due to the patients’ denial to undergo invasive angiography. DSA confirmed the findings of MRI/MRA, showing mostly a “flame-shaped” occlusion of the affected artery (Figure 1 and Figure 2), focal or multiple stenoses, and the beaded appearance in cases of FMD (Figure 4H,I). Abnormalities in the renal arteries were additionally demonstrated in four patients (Figure 4G). Neuroimaging findings are summarized in Table 2. Four patients were finally diagnosed with fibromuscular dysplasia (Figure 4), whereas cervical computed tomography (CT) showed elongated styloid processes bilaterally, indicative of Eagle syndrome in another patient (Figure 5D). This patient reported an influenza-like syndrome 15 days preceding neurological symptom onset.

Intravenous thrombolysis was administered to two patients (one received alteplase and the other one tenecteplase), whereas endovascular therapy was not performed in any case. All patients were treated with either antiplatelets (eight patients) and/or anticoagulation (three patients). Substantial or complete clinical recovery was observed in 10 out of 11 patients, while one patient experienced recurrent ischemic stroke during a mean follow-up period of 9 ± 4 months, prompting the addition of antiplatelet therapy to ongoing anticoagulation.

## 4. Discussion

In the present study, we investigated consecutive patients suffering from multiple cervical artery dissections. Utilizing high-resolution MRI vessel wall imaging systematically in patients with suspected cervical artery dissection or in those with isolated CAD enabled early detection of multiple dissections, even when asymptomatic. Thorough systemic screening identified underlying connective tissue disorders (FMD in four patients) and other vasculopathies (Eagle syndrome in one patient). Moreover, the detection of multiple CADs allowed a more careful risk stratification of subsequent ischemic stroke or stroke recurrence and necessitated a more intensive monitoring of the patient.

Notably, detection of asymptomatic cervical dissections means high-resolution MRI altered meaningfully our management strategy. First of all, this information prompted us to perform DSA in the vast majority of our patients, confirming fibromuscular dysplasia as the underlying cause of multiple cervical dissections in four patients. Other radiological markers were evaluated more thoroughly, leading also to diagnoses such as Eagle syndrome. Predisposing risk factors were investigated more thoroughly, facilitating their recognition and guiding patient counseling to prevent future exposure. Moreover, all our patients received more careful management of vascular risk factors, with consideration of the increased risk of dissection and/or ischemic event recurrence.

The mean age at diagnosis in our case series was 48 years, aligning with the age range documented in the literature, with a marginal female predominance [26]. Despite the established association between cervical trauma or chiropractic interventions and multiple CADs, this was not reflected in our series, with only a single patient reporting a history of trauma [3]. Intense physical exercise, childbirth, and low body mass index were among the additional risk factors recognized in our series [6,27,28]. Notably, none of the patients in our cohort were found to have hyperhomocysteinemia [29,30].

The most common clinical symptom in our cohort was headache and/or neck pain, followed by focal neurological signs attributed to cerebral infarcts. Pain, localized ipsilateral to the site of dissection, is noted to have a variable onset, developing after a delay of hours, days, or, in some instances, weeks, and has also been described as sudden, severe cervical pain, occasionally accompanied by Horner’s syndrome or headache. Intermittently, the headache may manifest with features resembling those of migraine, even mimicking status migrainosus. Furthermore, presentations resembling cluster headache have also been described [31,32]. Notably, intracranial dissections and/or subarachnoid hemorrhage were not documented in our cases [33]. These findings are in agreement with prior studies, highlighting the presence of cervical pain and headache in up to 66% and 71% of cases, respectively [34].

The results of our study, reporting that 36% of our patients had FMD as an underlying cause of their multiple dissections, are consistent with the existing literature, indicating that underlying connective tissue vasculopathies are more frequent in patients with multiple dissections [35]. Beyond FMD, other hereditary connective tissue disorders with well-documented vascular involvement not detected in our patients, in particular, including vascular Ehlers–Danlos syndrome, Marfan syndrome, osteogenesis imperfecta, and Loeys–Dietz syndrome, are associated with structural fragility of the arterial wall and have been implicated as predisposing factors for spontaneous CADs [7]. Recognition of these rare disorders is clinically relevant, as they not only influence the risk of recurrence and systemic vascular complications but also carry important implications for genetic counseling and long-term management [36,37]. Routine application of genetic testing, including panels targeting established connective tissue–related mutations in patients with spontaneous multiple CADs, warrants acknowledgment and additional recommendation.

Hypertension has also been identified as a predisposing factor for CAD, with available evidence supporting a causal association between higher systolic blood pressure and increased risk of CAD and early recurrence [38,39]. This association has been partially attributed to the underlying existence of FMD, which also affects the renal arteries, leading to hypertension [40]. In our case series, five out of eleven patients had a history of arterial hypertension, and in one of them, FMD was identified.

Since multiple CADs can occur simultaneously, the possibility of a transient post-infectious arteritis in a subset of patients has been reported. Inflammatory pathways activated during infection, together with the action of microbial agents, can lead to marked impairment of the vascular wall [41]. A more recent population-based analysis suggested an association between recent influenza-like illness and cervical artery dissection [5]. This has been documented in one of our patients, finally diagnosed with Eagle syndrome. Respiratory infections, accompanied by cough, may be severe enough to cause dissection, but this seems, however, very rare [42]. Neuroimaging-specific signs, including peri-arterial edema and attenuation of pericarotid fat, have been previously described as potential imaging biomarkers of inflammation in spontaneous dissections [43,44]. Nevertheless, these signs have not been identified in our cohort.

Traditional imaging methods, including carotid ultrasound, computed tomography angiography (CTA), MRA, and DSA, indirectly suggest abnormalities in the affected vessel wall [17,45]. Moreover, vessel wall abnormalities may be missed when luminal appearance is unremarkable [46]. In contrast, high-resolution MRI vessel wall imaging has demonstrated multiple advantages in the diagnosis of CAD [14,47,48]. This method provides, in a relatively short imaging time, full coverage of the head and neck blood vessels in a single acquisition [14]. Additionally, the 3D T1-weighted black-blood sequence, providing maximum surface reconstruction, allows the visualization of the length and structural characteristics of the intramural hematoma in a three-dimensional manner. This aids in detecting dissections, even in segments with tortuous arterial courses [49]. Finally, the T1-weighted black-blood sequence, as a safe and noninvasive method, represents an important tool for neuroimaging follow-up.

Characteristic high-resolution MRI vessel wall imaging signs include the intimal flap or double-lumen sign, intramural hematoma, arterial perivascular edema, and intramural thrombus. The strip-like signal between the true and false lumens or between the true lumen and the intramural hematoma is the direct sign of dissection, indicating active dissection and potential luminal compromise [50]. Intramural hematoma with the associated temporal evolution mechanism is due to the tearing of the arterial intima. This leads to the blood entry into the vessel wall, also called intimal tear type, or leads to the rupture of vasa vasorum [51].

In terms of stroke prevention among patients with CAD, there is inconsistent evidence regarding the safety and efficacy of anticoagulation compared with antiplatelet therapy [19,52]. The most recent systematic review and meta-analysis of the available literature, including also the largest observational study to date, has shown a superiority of anticoagulation over antiplatelets in reducing ischemic stroke; however, there is a higher risk of major bleeding [20,53]. This underscores the need for an individualized treatment strategy that balances the net clinical benefit of reducing ischemic stroke against the risk of bleeding and of randomized–controlled clinical trials and larger observational cohort studies to address this critical query. Similarly, in our cohort, treatment decisions were oriented toward the patient (e.g., anticoagulation was given to patients with either a known history of paroxysmal atrial fibrillation or stroke recurrence) and influenced by the physician’s judgment.

The present study has some limitations that should be acknowledged. First, the small sample size of our cohort (*n* = 11) combined with a relatively short follow-up time predisposes to selection bias and limits the feasibility of conducting in-depth statistical analyses to assess predictors of clinical manifestations, stroke recurrence, and clinical outcomes. Second, it was not possible to distinguish between multiple simultaneous dissections and early recurrent dissections in our cohort study. This would be meaningful in terms of recurrent cerebral ischemia risk assessment and stratification and probable treatment strategy differentiation, since early recurrent CADs have been associated with increased risk of stroke relapse [54]. Third, the absence of genetic testing for connective tissue disorders in our cohort precludes considering the diagnostic workup as comprehensive. Fourth, arterial tortuosity, which is elevated in patients with spontaneous CADs years after dissection occurrence and has been shown to be associated not only with the occurrence but also with long-term dissection recurrence, has not been systematically evaluated and quantified in our cohort [8,55,56]. Moreover, an individualized treatment approach leading to treatment heterogeneity prevents drawing firm conclusions on whether one treatment is superior to the other. Future, larger observational studies exploring the relationship between multiple dissections and long-term recurrence are warranted.

## 5. Conclusions

In conclusion, the present study emphasizes the potential role of specific high-resolution MRI sequences as a valuable tool for the detection of multiple CADs in young patients. The systematic application of these imaging techniques may not only enhance the sensitivity for identifying multiple CADS but also facilitate a more thorough evaluation for underlying connective tissue vasculopathies. Improved detection can contribute to more accurate risk stratification for ischemic stroke and may have important implications for individualized acute reperfusion and secondary prevention strategies. These findings support the integration of advanced MRI protocols into routine clinical practice for young patients presenting with suspected CAD.

## Figures and Tables

**Table 1:** Evidence on demographics, clinical manifestations, treatment, and outcome.

	Age	Sex	Medical History	History of Trauma	Baseline Clinical Presentation	Fibromuscular Dysplasia	Treatment	Outcome	Three-Month mRS
Pat. #1	55	F	Arterial hypertension	No	Sudden onset of right-sided Horner syndrome and left-sided hemiparesis with sensory deficits—NIHSS:4	No	Dual antiplatelet therapy (Clopidogrel 75 mg/day plus Acetylsalicylic acid 100 mg/day) for 21 days, followed by Clopidogrel 75 mg/day	Substantial clinical recovery—NIHSS: 0 at discharge	0
Pat. #2	61	F	Paroxysmal atrial fibrillation	No	Right-sided hemiparesis and hemihypesthesia, aphasia, dysarthria, right-sided hemianopsia and facial paresis. NIHSS: 9	No	Intravenous thrombolysis with alteplase. Anticoagulation with low-molecularweight heparin, followed by Rivaroxaban (20 mg/day)	Significant clinical recovery—NIHSS: 4 at discharge	2
Pat. #3	42	F	Unremarkable	Yes	Within 24 h following head and neck trauma, Horner syndrome and left-sided maxillofacial pain	No	Dual antiplatelet therapy (Clopidogrel 75 mg/day plus Acetylsalicylic acid 100 mg/day) for 21 days, followed by Clopidogrel 75 mg/day	Complete clinical recovery	0
Pat. #4	48	F	Anorexia nervosa, smoking	No trauma, symptoms onset after intense physical exercise	Left-sided cervicocranial pain, frequently mimicking cluster headaches. Nausea and vomiting	Yes	Dual antiplatelet therapy (Clopidogrel 75 mg/day plus Acetylsalicylic acid 100 mg/day) for 21 days, followed by Clopidogrel 75 mg/day.	Complete clinical recovery	0
Pat. #5	60	M	Arterial hypertension, hyperlipidemia	No	Horner syndrome right, headache, visual disturbances, dizziness, and gait imbalance. Symptoms onset 2 months ago.	NA	Clopidogrel 75 mg/day.	Complete clinical recovery	0
Pat. #6	42	M	Unremarkable	No	Left frontal headache, vision disturbances, left-sided sensory deficits	Yes (confirmed in DSA)	Dual antiplatelet therapy (Clopidogrel 75 mg/day plus Acetylsalicylic acid 100 mg/day) for 21 days, followed by Clopidogrel 75 mg/day	Substantial clinical recovery—NIHSS: 0 at discharge	0
Pat. #7	41	M	Arterial hypertension, hyperlipidemia, smoking	No	Sudden onset of right-sided hemiparesis with dysarthria/aphasia	Yes	Dual antiplatelet therapy (Clopidogrel 75 mg/day plus Acetylsalicylic acid 100 mg/day) for 21 days, followed by Clopidogrel 75 mg/day	Substantial clinical recovery—NIHSS: 0 at discharge	0
Pat. #8	57	M	Arterial hypertension, papillary thyloid carcinoma 40 years ago, hypothyroidism	No	Sudden onset of right-sided hemiparesis and hemihypesthesia, left-sided amaurosis and dysarthria with mixed aphasia. NIHSS: 19 on admission	NA	Intravenous thrombolysis with tenecteplase. Dual antiplatelet therapy (Clopidogrel 75 mg/day plus Acetylsalicylic acid 100 mg/day) for 21 days, followed by Clopidogrel 75 mg/day	Substantial clinical recovery—NIHSS: 3 at discharge	0
Pat. #9	40	F	Hyperlipidemia, smoking	No	Transient ischemic attack with right-hand weakness, followed by aphasia and right-sided hemiparesis and hemihypesthesia	No	Anticoagulation with low-molecular-weight heparin, followed by dual antiplatelet therapy (Clopidogrel 75 mg/day plus Acetylsalicylic acid 100 mg/day) after follow-up	Substantial clinical recovery—NIHSS: 0 at discharge	0
Pat. #10	51	M	Hyperlipidemia, sleep apnoea, arterial hypertension, previous smoking	No	Right0sided cervicocranial pain, visual disturbances, mixed aphasia, gait disturbances NIHSS:3	No	Anticoagulation with low-molecular-weight heparin	Recurrent ischemic stroke, frontal left Antiplatelet therapy (Clopidogrel 75 mg/day) was added to the already administered anticoagulation NIHSS:7	1
Pat. #11	33	F	Childbirth with cesarean section one month earlier	No	Left-sided headache, transient ischemic attack with right-sided hemiparesis and hemihypesthesia	Yes	Dual antiplatelet therapy (Clopidogrel 75 mg/day plus Acetylsalicylic acid 100 mg/day) for 21 days, followed by Clopidogrel 75 mg/day	Complete clinical recovery	0

Abbreviations: DSA: digital subtraction angiography; F: female; M: male; mRS: modified Rankin Scale; NA: not available; NIHSS: National Institutes of Health Stroke Scale; Pat.: patient.

**Table 2:** Neuroimaging findings.

	MRI Findings	MRA Findings	3D T1 SPACE Findings	DSA Findings	Affected Cervical Arteries	Number of Dissected Vessels	Affected Renal Arteries
Pat. #1	Acute right MCA infarction	Right internal carotid “flame-like” occlusion (≈1 cm above the bifurcation)	Intramural hematoma along the distal cervical and proximal petrous artery segments and mural hematoma in the intraforaminal segment of the right (V2) vertebral artery	Confirmed MRA findings	Symptomatic right ICA and asymptomatic right V2 dissections	2	No
Pat. #2	Acute left MCA infarction	Left internal carotid “flame-like” occlusion (≈1 cm above the bifurcation)	Intramural hematoma along the distal cervical and proximal petrous artery segments of the left ICA. and Mural hematoma in the intraforaminal segment of the right (V2) vertebral artery	Confirmed MRA findings	Symptomatic left ICA dissection and asymptomatic right V2 dissection	2	No
Pat. #3	No ischemic lesions	Stenosis along the distal cervical and proximal petrous segments of the left ICA with dissecting pseudoaneurysm along the proximal segment of the left ICA dissection	Intramural hematoma along the distal cervical and petrous segments of the ICA bilaterally	Confirmed MRA findings	Asymptomatic right ICA and symptomatic left ICA dissections	2	No
Pat. #4	No ischemic lesions	Stenosis along the distal cervical segment of the right ICA and the distal cervical and proximal petrous segments of the left ICA Reduction in the diameter at the peripheral half of the foraminal (V2) segment of the right vertebral artery	Intramural hematoma along the cervical segments of the ICA bilaterally	Confirmed MRA findings	Right ICA and left ICA dissections and highly suspected right V2 segment dissection	2	Yes—abnormal findings along the right renal artery
Pat. #5	No ischemic lesions	Right ICA occlusion. Occlusion along the V4 segment of the left vertebral artery. Sequential stenosis along the V4 segment of the right vertebral artery	intramural hematoma along the cervical and proximal petrous artery segments of the right ICA and Mural hematoma in the V4 segments of the right and left vertebral arteries	DSA was recommended but declined by the patient	Right ICA dissection. Dissections along the right and left V4 segments of the vertebral arteries	3	No
Pat. #6	Chronic ischemic lesion in the right inferior frontal gyrus	Focal stenoses along the distal cervical segments of the right and left ICA	Intimal flap along the distal cervical segment of the right ICA Intramural hematoma along the distal cervical segment of the left ICA	DSA confirmed the findings	Right and left ICA dissections	2	Yes—wall abnormalities along the right renal artery, indicative of fibromuscular dysplasia
Pat. #7	Yes—multiple ischemic lesions in different arterial territories	Stenoses along the cervical segments of the right and left ICA. Characteristic “string-of-beads” appearance along the cervical segment of the left ICA	Intramural hematoma along the cervical segment of the right ICA. Intramural hematoma along the cervical segment of the left ICA, combined with the typical beaded appearance	DSA confirmed the findings and also revealed a focal stenosis of the right renal artery	Asymptomatic right and symptomatic left ICA dissections	2	Yes—focal stenosis of right renal artery
Pat. #8	Yes—acute ischemic stroke in the territory of the MCA artery	Significant stenosis along the cervical segment of the left ICA	Focal intramural hematoma along the cervical segment of the right ICA Intramural hematoma along the cervical and proximal petrous segments of the left ICA	DSA was recommended but declined by the patient	Asymptomatic right and symptomatic left ICA dissections	2	No
Pat. #9	Acute left MCA infarction	Right internal carotid near occlusion along the cervical segment of the artery Left internal carotid “flame-like” occlusion (≈0.5 cm above the bifurcation)	Intramural hematoma along the cervical and proximal petrous artery segments of the right and left ICAs	Confirmed MRA findings	Asymptomatic right and symptomatic left ICA dissections	2	No
Pat. #10	Bilateral multiple ischemic lesions in different arterial territories	Stenosis along the distal cervical and proximal petrous segments of the right ICA. Stenosis along the distal cervical and proximal petrous segments of the right ICA.	Chronic intramural hematoma along the distal cervical segment of the right ICA. Intramural hematoma along the cervical and proximal petrous artery segments of the left ICA	Confirmed MRA findings CT revealed elongated styloid processes bilaterally, indicative of Eagle syndrome	Symptomatic right and left ICA dissections	2	No
Pat. #11	Acute multiple left MCA infarctions	Stenosis along the distal cervical and proximal petrous segments of the right ICA with dissecting pseudoaneurysm along the proximal segment of the right ICA dissection Left internal carotid “flame-like” occlusion, above the bifurcation	Intramural hematoma along the cervical segment of the right ICA Intramural hematoma along the cervical and proximal petrous artery segments of the left ICA	Confirmed MRA findings Mild wall abnormalities and segmental dilatations of the lumen were also observed along the V2 vertebral artery segments bilaterally	Asymptomatic right ICA dissection and symptomatic left ICA dissection	2	Mild wall abnormalities of the trunk of the right renal artery, without stenosis, indicative of fibromuscular dysplasia

Abbreviations: CT: computed tomography; DSA: digital subtraction angiography; ICA: internal carotid artery; MCA: middle cerebral artery; MRI: magnetic resonance imaging; MRA: magnetic resonance angiography; Pat.: patient; SPACE: sampling perfection with application-optimized contrast using different flip-angle evolutions.

## Data Availability

All data needed to evaluate the conclusions in the paper are present in the main manuscript. Additional data related to this paper may be requested from the corresponding author upon reasonable request.
